# Blood Pressure J-Curve: Current Concepts

**DOI:** 10.1007/s11906-012-0314-3

**Published:** 2012-10-04

**Authors:** Maciej Banach, Wilbert S. Aronow

**Affiliations:** 1 Department of Hypertension, Chair of Nephrology and Hypertension, Medical University of Lodz, Lodz, Poland; 2 Department of Medicine, New York Medical College, Valhalla, NY USA; 3 Department of Hypertension, WAM University Hospital in Lodz, Medical University of Lodz, Poland. Zeromskiego 113, 90-549 Lodz, Poland

**Keywords:** Blood pressure, J-curve, High-risk populations, Cardiovascular risk, Diabetes, Coronary artery disease, CAD, Elderly, Left ventricle hypertrophy, LVH, Hypertension, Hypotensive therapy, Antihypertensive treatment

## Abstract

The blood pressure (BP) J-curve debate started in 1979, and we still cannot definitively answer all the questions. However, available studies of antihypertensive treatment provide strong evidence for J-shaped relationships between both diastolic and systolic BP and main outcomes in the general population of hypertensive patients, as well as in high-risk populations, including subjects with coronary artery disease, diabetes mellitus, left ventricular hypertrophy, and elderly patients. However, further studies are still necessary in order to clarify this issue. This is connected to the fact that most available studies were observational, and randomized trials did not have or lost their statistical power and were inconclusive. Perhaps only the Systolic Blood Pressure Intervention Trial (SPRINT) and Optimal Blood Pressure and Cholesterol Targets for Preventing Recurrent Stroke in Hypertensives (ESH-CHL-SHOT) will be able to finally answer all the questions. According to the current state of knowledge, it seems reasonable to suggest lowering BP to values within the 130–139/80–85 mmHg range, possibly close to the lower values in this range, in all hypertensive patients and to be very careful with further BP level reductions, especially in high-risk hypertensive patients.

## Introduction

Arterial hypertension is the number one direct cause of death in the world and the third cause of disability. Through the metabolic syndrome, it is closely related to other major health threats of modern society such as inactivity, obesity, hyperglycemia and dyslipidemia [1–3]. About 54 % of strokes and 47 % of ischemic heart disease cases worldwide are attributable to high blood pressure (BP), and hypertension is present in approximately 69 % of patients with a first myocardial infarction (MI), in approximately 77 % of patients with a first stroke, in approximately 74 % of patients with chronic heart failure (HF) and in 60 % of patients with peripheral arterial disease [3–5].

In 2009 the reappraisal of the guidelines of the European Society of Hypertension (ESH) addressed the issue of the so-called J-curve (or U-curve) and the clinical implications stemming from this phenomenon for the first time [6, 7]. The recommendations also suggest lowering the systolic BP (SBP)/diastolic BP (DBP) to values within the 130–139/80–85 mmHg range, possibly close to the lower values in this range, in all hypertensive patients [6]. This was a result of the publication of several important studies and *post hoc* analyses concerning intensive BP lowering in patients from high-risk groups, including especially hypertensive subjects with diabetes (DM) and coronary artery disease (CAD) [8–10]. The recent data indicate that problems with intensive BP reduction can also be present in the elderly, in patients with left ventricle dysfunction and after stroke/transient ischemic attack (TIA) [11–13]. These data challenge the current belief that, in patients at cardiovascular (CV) risk, a decrease in BP to below 120/70-75 mmHg is associated with a reduction of CV events; on the contrary, they suggest an increase in the risk of adverse CV events [6–13].

This review aims to answer questions about the mechanisms involved in the J-curve phenomenon, to determine the patient groups that may potentially be at the highest risk and finally to determine whether there is strong enough evidence to confirm that there is such a phenomenon in hypertensive patients.

## Search Strategy

We searched using the electronic databases MEDLINE (1966–August 2012), EMBASE and SCOPUS (1965–August 2012), and DARE (1966–August 2012). Additionally, abstracts from national and international cardiovascular meetings were searched. Where necessary, the relevant authors were contacted to obtain further data. Retrospective studies, as well as small studies with fewer than 100 patients, were excluded from the review. The main data search terms were: blood pressure, diabetes, hypertension, intensive (aggressive) hypotensive therapy, J-curve, therapy and treatment.

## What is the J-curve Phenomenon?

The discussion on the J-curve (U-curve) phenomenon started in 1979 when Stewart presented the results of studies conducted in 169 patients with severe hypertension [14]. He noticed that the relative risk of MI was over five-fold higher in individuals who had achieved a DBP reduction <90 mmHg compared with a BP in the range of 100–109 mmHg [10, 14]. Subsequent reports, published in the 1980s and 1990s, confirmed those observations [10, 15]. Therefore, the J-curve phenomenon is defined as the shape of the relationship between BP and the risk of CV morbidity and mortality, which means that the risk of CV events may increase at both too high and too low levels of BP. Hypertension specialists are also in agreement that there is a lowest value of BP (nadir), which represents a point at which BP is too low to maintain perfusion of vital organs, particularly the heart [10].

Initially, the J-curve phenomenon was described for DBP in patients with CAD [10, 16]. Most coronary perfusion occurs during diastole, and DBP lowering results in low coronary perfusion pressure and dilation of coronary microvessels [10, 17]. After the maximal vasodilation has been achieved, further reduction of perfusion pressure results in decreased coronary blood flow. In patients who have stenotic lesions of large coronary arteries [e.g., with DM, CAD and left ventricle hypertrophy (LVH), where myocardial O_2_ consumption is essentially increased), an upward shift of perfusion pressure is required to maintain distal flow past the stenosis, and therefore there is much less tolerance of DBP reduction [17, 18]. Therefore, a further fall in DBP in these patients may lower the coronary perfusion pressure to a critical level, intensifying ischemia and potentially causing a CV event [17]. Finally, it is also important to mention that low coronary flow is associated with increased blood viscosity and platelet adhesiveness, predisposing to intracoronary thrombosis and MI [10, 17–20].

Recently, there have also been some data on the J-curve phenomenon for SBP, although the majority of studies focus on DBP. This is connected to the fact that only in isolated cases is it possible to achieve SBP values low enough to potentially pose any threat to the patient. However, it is worth noting that since lowering the DBP is closely associated with lowering SBP, the problem may arise of patients with high pulse pressure while their SBP values are still high but DBP low. The current recommendation is that the SBP should still be lowered without concern for the DBP, since it is the high SBP that is most predictive in terms of CV event occurrence [10, 21, 22].

Despite increasing evidence for the J-curve, there has also been a debate on possible sources of bias concerning this phenomenon (this has been previously discussed [10]), which is especially connected to the intrinsic biases in the identification of a J-shape of risk factor relationships [10]. Hypertension as a primary risk factor (and the beginning of the ‘cardiovascular disease continuum’) may be negatively confounded with other residual risk factors. Thus, it is not surprising that the J-curve phenomenon might also occur in the analyses of cholesterol and low density lipoprotein cholesterol (LDL-C) (‘lipid paradox’), body mass index (BMI; ‘obesity survival paradox’), glucose or uric acid [10, 23, 24]. One should note that if this risk confounding combines with effect modification between the primary and residual risk factors, then the aggregate effect is a nonlinear distortion of the risk factor relation, which may produce an apparent threshold or J-curve relationship [10]. This was confirmed in a large collaborative meta-analysis of 102 prospective studies that enrolled a total of almost 700,000 persons without a history of vascular disease or diabetes at baseline, where a J-shaped relationship was observed both between SBP and coronary heart disease (CHD; nadir: 130 mmHg) and between CV events and other major risk factors, including fasting blood glucose and total and non-high-density lipoprotein (HDL) cholesterol [25].

## Optimal BP in High CV Risk Patients

Apart from CAD and DM patients, the J-curve phenomenon has been observed in studies including high-risk hypertensive patients. On the other hand, there are also studies that did not confirm the phenomenon. In the Swedish Behandla Blodtryk Bättre (BBB)–Treat Blood Pressure Better Study [26], the authors compared the effects of intensive hypotensive therapy aimed at a reduction of DBP to <80 mmHg with the maintenance of DBP values in the range of 90-100 mmHg (standard treatment) in hypertensive patients and did not find an increased occurrence of adverse effects in patients with DBP <80 mmHg [26]. In the Hypertension Optimal Treatment (HOT) study [27], the patients (*n* = 18,790) were randomized into three groups that aimed at achieving DBP values ≤90, ≤85 or ≤80 mmHg. The authors showed that the lowest risk of CV events was observed at a DBP of 82.6 mmHg, while a decrease of DBP below this level had no effect on the reduction of risk of CV complications. The study also did not demonstrate any increase in CV event incidence in the group of patients with DBP <70 mmHg [27]. At this point it is, however, necessary to emphasize that the HOT study had some important limitations (it has been previously discussed [10]). Due to the lack of detailed data on the group of 3,080 patients with cardiac ischemia, the final conclusion concerning the safety of intensive DBP lowering was called into question [10, 28]. The inclusion of those data suggested the existence of a J-curve relationship between the incidence of CV events (MI) and the DBP values (<80 mmHg) in patients with CAD and the absence of such a relationship in a group of individuals not suffering from CAD [10, 28, 29] (Table 1).

**Table 1:** Summary of the most important studies in which a J-curve relationship was observed between either DBP or SBP and adverse outcomes

Study	Year	No. of participants (*n*)	High- risk patients	CAD	DM	LVH	Elderly	CKD	J-curve point, DBP, mmHg	J-curve point, SBP, mmHg
D'Agostino et al. (Framingham Heart Study) [16]	1991	5,209	Yes	Yes	-	-	-	-	75	-
Systolic Hypertension in The Elderly Program (SHEP) study [56]	1991	4,736	Yes	-	-	-	Yes	-	70 (55)^1^	-
Hypertension Optimal Treatment (HOT) Study [27]	1998	3,080	Yes	Yes	-	-	-	-	80	-
Vokó et al. (The Rotterdam Study) [55]	1999	7,983	Yes	-	-	-	Yes	-	65	-
Pastor-Barriuso et al. (Second National Health and Nutrition Examination Survey) [57]	2003	7,830	-	-	-	-	Yes	-	80-90	-
International Verapamil SR-Trandolapril Study (INVEST) [36]	2003	22,576	-	Yes	-	-	-	-	84	119
Systolic Hypertension in Europe (Syst-Eur) Trial [58]	2004	4,583	Yes	Yes	-	-	Yes	-	70	-
Valsartan Antihypertensive Long-term Use Evaluation (VALUE) trial [30]	2004	15,245	Yes	-	-	-	Yes	-	78	120–130
Irbesartan Diabetic Nephropathy Trial (IDNT) [47]	2005	1,590	-	-	Yes	-	-	Yes	85	120
Oates et al. [62]	2007	4,071	-	-	-	-	Yes	-	89	139
Ongoing Telmisartan Alone and in Combination with Ramipril Global Endpoint Trial (ONTARGET) [32]	2009	25,588	Yes	Yes	Yes	-	-	-	72	126–130
Treating to New Targets (TNT) [37]	2009	10,001	Yes	Yes	-	-	-	-	79.8 (60-70)^2^	140 (110-120)^2^
Agarwal et al. [71]	2009	218	-	-	-	-	-	Yes	70	-
PRavastatin Or atorVastatin Evaluation and Infection Therapy-Thrombolysis In Myocardial Infarction (PROVE IT-TIMI) 22 trial [39••]	2010	4,162	Yes	Yes	-	-	-	-	70	110
International Verapamil SR-Trandolapril Study (INVEST) [43••]	2010	6,400	-	Yes	Yes	-	-	-	-	115
International Verapamil SR-Trandolapril Study (INVEST) [63•]	2010	2,180	-	Yes	-	-	Yes	-	70	140
Action to Control Cardiovascular Risk In Diabetes – Blood Pressure Arm (ACCORD-BP) [44••]	2010	4,733	Yes	-	Yes	-	-	-	-	120 (119.3)^3^
Ogihara et al. [61]	2011	1,500	-	-	-	-	Yes	-	-	120
Ongoing Telmisartan Alone and in Combination with Ramipril Global Endpoint Trial (ONTARGET) [33]	2011	12,554	Yes	Yes	Yes	-	-	-	80	130
Digitalis Investigation Group (DIG) [66•]	2011	7,788	-	-	-	Yes	-	-	-	120
Beta-Blocker Evaluation of Survival Trial (BEST) [67]	2011	2,706	-	-	-	Yes	-	-	-	
Secondary Manifestations of Arterial Disease (SMART) study [40••]	2012	5,788	Yes	Yes	-	-	-	-	82	143
Losartan Intervention For Endpoint reduction in hypertension study (LIFE) [65]	2012	9,193	-	-	-	Yes	-	-	-	130
Vamos et al. [50•]	2012	126,092	-	Yes	Yes	-	-	-	70	110

*CAD* coronary artery disease; *DM* diabetes mellitus; *LVH* left ventricular hypertrophy; *CKD* chronic kidney disease; *DBP* diastolic blood pressure; *SBP* systolic blood pressure

^1^ The relative risk of composite cardiovascular events was close to two-fold greater for DBP <55 mmHg; ^2^ For the primary endpoints; ^3^ The SBP level below which an increase in therapy-related adverse events (orthostatic hypotension, hyperkalemia, syncope, bradycardia, arrhythmia or renal function impairment) was observed

In the Valsartan Antihypertensive Long-term Use Evaluation (VALUE) trial [30], patients aged ≥50 years with treated or untreated arterial hypertension, with high CV risk, were included and randomized to two parallel arms, receiving either valsartan (80-160 mg/day) or amlodipine (5-10 mg/day). The lowest risk of CV events was observed in the 120-130 mmHg SBP range (nadir), while a further reduction was associated with a significant increase in cardiac complications (CVD events). On the other hand, it must be emphasized that there were only a few people at the lower end of SBP values [30, 31] (Table 1).

**Fig. 1:**
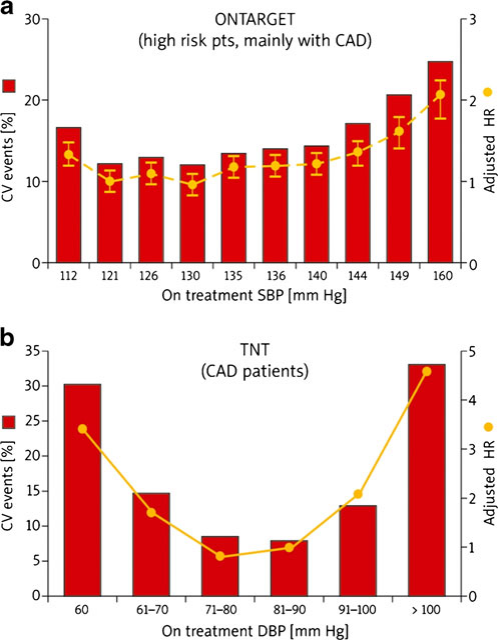
J-curve in the **(a)** ONTARGET ([10] modified according to [32, 33]) and **(b)** TNT ([10] modified according to [37]) studies

Similar results were obtained in the Ongoing Telmisartan Alone and in Combination with Ramipril Global Endpoint Trial (ONTARGET) [32], where 25,588 high-risk patients with known atherosclerotic disease or diabetes with vascular damage (9,603 patients) were included. The authors showed that achieving a significant reduction of SBP >130 mmHg was associated with a significantly higher risk of the occurrence of a composite endpoint comprising CV mortality (nadir: 130 mmHg) and MI (nadir: 126 mmHg). For any level of SBP achieved, the highest risk of a CV event or stroke was seen in participants with the DBP ≤72 mmHg [32]. The secondary analysis of ONTARGET compared the benefits and risks of treatment aiming to reach BP targets of <140/90 vs. <130/80 mmHg for cardiovascular complications (CVD, renal events and stroke) [33]. The authors showed that CV events were reduced by increasing the frequency of BP control to <140/90, but not to <130/80 mmHg. At the same time, no J-curve relationship was observed between the lowering of SBP and an increase in stroke risk (achievement of lower BP goals appeared to be useful in stroke prevention) [33, 34] (Fig. 1a and Table 1).

The results of the VALUE and ONTARGET trials were not confirmed in the Studio Italiano Sugli Effetti CARDIOvascolari del Controllo Della Pressione Arteriosa SIStolica (CARDIO-SIS) study [35], which aimed at evaluating whether strict control of SBP (target value <130 mmHg; *n* = 558), as compared to the standard one (target value <140 mmHg; *n* = 553) did reduce the risk of LVH and serious CV events in patients with hypertension but no diabetes and with high CV risk [35]. After 2 years of follow-up, in the strict control group as compared to the typical control, there was a significantly lower risk of total CV events, cardiac revascularization, recent atrial fibrillation and LVH, as well as comparable risk of total death, MI, hospitalization due to HF, stroke or transient ischemic attack (TIA). The results of the CARDIO-SIS study did not confirm the J-shaped relationship; however, it is important to remember the limitations of this trial: its relatively small size, short duration of follow-up and use of an intermediate endpoint (ECG-LVH) as the primary outcome [10, 17, 35].

## J-curve in CAD Patients

Patients with CAD are at increased risk of ischemic events beyond a certain DBP [13, 17–20]. In the Framingham Heart Study (FHS) [16], the authors observed an age- and sex-independent J-curve relation for DBP and CHD deaths in patients with MI (but not for low-risk subjects without MI), which was independent of left ventricular function and antihypertensive treatment [16]. These observations have been confirmed in many subsequent trials, the most important one being the International Verapamil SR-Trandolapril Study (INVEST) [36], where CAD patients undergoing intensive hypotensive treatment because of arterial hypertension (verapamil 240 mg/day or atenolol 50 mg/day, with the possibility of adding trandolapril at a starting dose of 2 mg/day as a second-line drug) were included. The authors found a J-shaped relationship with a nadir of 119/84 mmHg (129/74 mmHg after adjustment for time to primary outcome) between both SBP and DBP and all-cause mortality and MI in both treatment groups. DBP lowering to <80 mmHg resulted in a significant increase in the risk of MI as compared with patients with higher DBP. However, the risk of MI was also observed in individuals with DBP <60 mmHg (as high as 14 %; for comparison, for DBP ≥110 mmHg it rose by 13 %) [8, 36] (Table 1).

The Treating to New Targets (TNT) [37] study evaluated the impact of intensive hypolipidemic treatment (atorvastatin 80 vs. 10 mg) on the occurrence of a first serious CV event in patients with stable CHD and a baseline level of LDL-C <130 mg/dl (3.4 mmol/l). The final analysis revealed a significant correlation between CV events (all-cause mortality, CV mortality, nonfatal MI and stroke) and an excessive lowering of DBP (nadir: 79.8 mmHg) and SBP (nadir: 140 mmHg) (*p* < 0.0001 for all). However, the risk of occurrence of a primary endpoint rose once the SBP reached 110 mmHg and DBP 60 mmHg [hazard risk (HR) 3.1 and 3.3, respectively] [10, 37] (Fig. 1b and Table 1). This was also confirmed in the subanalysis of the TNT trial, which showed that in patients with CAD, a low BP (<110-120/<60-70 mmHg) portended an increased risk of future CV events (except stroke). The authors also noted that for the BP level (nadir) of 146.3/81.4 mmHg the risk of CV events was the lowest [38]. It is worth noting that this study involved on-treatment BP levels and included a population that was on statins with aggressive CV factor modification [17, 38] (Table 1).

In the analysis of the PRavastatin Or atorVastatin Evaluation and Infection Therapy-Thrombolysis In Myocardial Infarction (PROVE IT-TIMI) 22 trial, the authors evaluated 4,162 patients with acute coronary syndrome (ACS) randomized to pravastatin 40 mg or atorvastatin 80 mg [10, 39••]. A nonlinear Cox proportional hazards model showed a nadir of 136/85 mmHg at which the incidence of primary outcome was lowest. The curve was relatively flat for systolic BPs of 110 to 130 mmHg and diastolic BPs of 70 to 90 mmHg. On the basis of these results, the authors suggest that low BP, especially <110/70 mmHg, may be dangerous for patients with ACS [10, 39••] (Table 1).

These J-curve data from randomized trials are supported by findings of the recently published Secondary Manifestations of Arterial Disease (SMART) study [40••]. A total of 5,788 patients with symptomatic vascular disease were followed up for the occurrence of new vascular events (MI, stroke or vascular death) and all-cause mortality. During a median of 5.0 years, 788 patients experienced a new vascular event, and 779 died. The relationship between mean baseline SBP, DBP, or pulse pressure and the occurrence of vascular events followed a J-curve with increased event rates above and below the nadir of 143/82 mmHg. A similar nonlinear relationship was found for DBP and all-cause mortality [40••] (Table 1).

Very interesting results were observed in the most recent meta-analysis aiming to evaluate the BP targets in patients with CAD [41]. Fifteen randomized clinical trials enrolling 66,504 participants were included. The authors found that the intensive BP lowering group (≤135 mmHg) was associated with a 15 % decrease in HF rate and 10 % decrease in stroke rate, and the more intensive BP lowering group (≤130 mmHg) was associated with a reduction in MI and angina pectoris. They concluded that in patients with CAD, intensive SBP control to ≤135 mmHg and possibly to ≤130 mmHg was associated with a modest reduction in stroke and HF, but at the expense of hypotension (10.5 % risk) [41]. These results are interesting, but they add very little to the current knowledge on the J-curve in CAD hypertensive patients, as the authors did not analyze the outcomes associated with the reduction of BP levels below 120 or even 110 mmHg, they focused only on SBP, and finally the included studies were very heterogeneous, with different patients, therapies, study durations and endpoints [41].

## Optimal BP Level in Diabetic Patients

Diabetes increases the risk of CVD almost three-fold irrespective of the SBP values. However, the current ESH guidelines (2009) changed the hitherto recommended targeted level of BP in DM patients, i.e., SBP < 130 mmHg, and suggested that the only confirmed therapeutic target was lowering the BP to below 140/90 mmHg (optimally in the range 130-139/80-85 mmHg) [6, 10, 42].

The above has been supported by recent studies, including a retrospective analysis of the INVEST study [43••] in which the patients were divided into three groups depending on the achieved BP: (1) those who had not reached the control level (SBP ≥140 mmHg), (2) those who had reached the standard control level (SBP <130-140 mmHg) and (3) those on intensive BP control (SBP ≤130 mmHg) [10, 42]. In patients with non-controlled BP, the risk of death, MI or stroke was as much as 50 % higher compared to those with controlled BP (HR 1.46; *p* < 0.0001). The authors also observed increased risk of death due to any cause – about 8 % after 30 months and 5 years after the study [adjusted HR: 1.20 (*p* = 0.06) and 1.15 (*p* = 0.04), respectively] in patients with intensively controlled BP. Additional analyses revealed that this risk was caused by a higher incidence of death in patients with SBP below 115 mmHg [43••]. Although the above data could have been burdened with some errors (i.e., the lack of initial division into groups with different target BP values or lack of information on how the patients with the lowest SBP had been managed), they confirm the risk associated with excessively intensive lowering of BP in DM patients [10] (Table 1).

The Action to Control Cardiovascular Risk In Diabetes – Blood Pressure Arm (ACCORD-BP) study [44••] was designed to evaluate the impact of treatment aimed at intensive lowering of SBP to <120 mmHg (compared to standard therapy) on the incidence of CV events in 4,733 DM patients. The study enrolled high-risk DM patients: aged ≥40 and with coexisting CVD; or aged ≥55 with marked atherosclerosis, albuminuria, and LVH; or with at least two risk factors for CVD: dyslipidaemia, arterial hypertension, smoking or obesity [44••]. After a year of treatment, the mean SBP was 119.3 mmHg in the group managed intensively and 133.5 mmHg in the group on standard therapy, while the mean DBP values were 64.4 and 70.5 mmHg, respectively [10, 44••]. The primary endpoint, comprising nonfatal MI or stroke, or death due to CV causes, occurred in 445 patients (1.87 % per year in the group on intensive treatment compared with 2.09 % of those on standard therapy; *p* = 0.20). In addition, there were 294 deaths due to any cause (1.28 % in the intensive therapy group vs. 1.19 % in the standard treatment group; *p* = 0.55) and 118 due to cardiovascular causes (0.52 % vs. 0.49 %, respectively; *p* = 0.74) [10, 44••]. The incidence of stroke was significantly higher in the group receiving standard treatment (0.53 % vs. 0.32 %; *p* = 0.01); a similar relationship was found for nonfatal stroke (0.30 % vs. 0.47 %; *p* = 0.03) [10, 44••]. It was concluded that intensive hypotensive therapy did not significantly reduce the incidence of primary endpoints or the majority of secondary endpoints; however, it was associated with a significant reduction in the total number of strokes (by 41 %; HR 0.59; 95 % CI, 0.39–0.89; *p* = 0.03) and nonfatal strokes (by 37 %) [10, 44••, 45] (Table 1).

As was previously discussed [10], the interpretation of ACCORD-BP results is neither easy nor obvious, since the group receiving standard treatment exhibited a lower (by over 50 %) rate of events than expected initially, and in the intensive therapy group, the incidence of adverse complications of treatment (orthostatic hypotension, hyperkalemia, syncope, bradycardia, arrhythmia or renal function impairment) was significantly increased (3.3 % vs. 1.3 %) [44••, 45]. Two important conclusions can be drawn on the basis of the ACCORD study: (1) it is important to define the group of patients in whom significant BP reduction could be particularly dangerous and – on the other hand – those with a high risk of stroke who could benefit most from an intensive hypotensive therapy, and (2) lowering of SBP to below 115 mmHg (also as a result of INVEST study analysis) may be dangerous, and hence the absolute safety limit in DM patients should be accepted as SBP in the range of 110-115 mmHg [10, 43••, 44••, 45, 46].

Similar results were observed in two other studies: in the Irbesartan Diabetic Nephropathy Trial (IDNT) [47], DBP <85 mmHg was associated with a trend to an increase in all-cause mortality, a significant increase in MI, but a decreased risk for stroke [47] (Table 1); in the Appropriate Blood Pressure Control in Diabetes-Normotension (ABCD-NT) trial [48], SBP <130 mmHg was not associated with a benefit in the primary outcome (renal dysfunction) or any other CV outcome. The active group participants did benefit from a significant reduction in stroke. A lack of benefits of lowering the SBP level <130 mmHg in patients with diabetes was also observed in the recent analysis of the ONTARGET trial [49].

The most recent trial not only confirmed the lack of benefits of lowering the SBP below 130 mmHg, but also the J-shaped relationship in DM patients [50•]. A total of 126,092 adult patients (age ≥18 years) from the United Kingdom General Practice Research Database with a new diagnosis of type 2 diabetes were included. In patients with CVD, tight control of SBP (<130 mmHg) and DBP (<80 mmHg) was not associated with improved survival. Low BP was also associated with an increased risk of all-cause mortality [50•]. Compared with patients who received usual control of SBP (130-139 mmHg), the HR of all-cause mortality was 2.79 (95 % CI 1.74-4.48, *p* < 0.001) for SBP at 110 mmHg. Compared with patients who received usual control of DBP (80-84 mmHg), the HRs were 1.32 (1.02-1.78, *p* = 0.04) and 1.89 (1.40-2.56, *p* < 0.001) for DBP at 70-74 mmHg and lower than 70 mmHg, respectively. Similar associations were found in people without CVD [50•] (Table 1).

## Optimal Blood Pressure in the Elderly

In Europe the average life expectancy at birth has risen from around 45 years in the 1900s to 65.6 years in 1950-1955 to 75.1 years in 2005-2010, and in the EU (European Union) 27, it is expected to rise to 85.3 years for women and 80.0 years for men [2, 51]. The number of Europeans aged 65+ is expected to increase by 45 % between 2008 and 2030, and even further to over 30 % of the population by 2060 [2, 51–53]. The same situation concerns the US, Japan and other countries. Therefore, currently we face the challenge not only to increase the length of life, but also to improve the quality of therapy and in consequence the quality of life in the elderly. Recently, there has been extensive discussion on the effective treatment of hypertension in the elderly population. This is connected to the fact that despite the available data we still do not know what the optimal BP levels are in these patients (or whether we can observe a J-curve relationship), which drugs are the most effective and have the fewest therapy-associated adverse events, and how the hypertension therapy can really prevent complications, reduce mortality and improve quality of life [2, 51, 54].

The studies published in the 1990s provided evidence of J-shaped relationships between DBP and CVD outcomes in elderly populations [27, 28, 55, 56]. The main aim of both the observational Rotterdam Study [55] and the Systolic Hypertension in the Elderly Program (SHEP) study [56] was the assessment of whether long-term application of hypotensive treatment in the elderly with isolated systolic hypertension decreases the incidence of stroke. The authors of both studies revealed a high risk of stroke occurrence also in the presence of low DBP values <65 mmHg [10, 55, 56]. The SHEP study also demonstrated that the intensive lowering of DBP was a factor predisposing to CV events. The relative risk of composite CV events was significantly higher for DBP values <70 mmHg and close to two-fold greater for DBP <55 mmHg [10, 56]. The association between low DBP and increased CV risk was also observed in 7,830 white and African-American men and women over 65 years of age who were participants in the Second National Health and Nutrition Examination Survey (NHANES II). Decrease in DBP below approximately 80 to 90 mmHg was significantly associated with increasing all-cause and CV mortality [10, 57] (Table 1).

The Systolic Hypertension in Europe (SYST-EUR) study [58] enrolled patients aged ≥60 years not exhibiting symptoms of dementia and with SBP and DBP values of 160-219 mmHg and ≤95 mmHg, respectively. The study revealed that lowering of the DBP to ≥55 mmHg did not result in an increase in death rates due to CV events, while it could potentially be associated with a higher death rate due to non-cardiac causes (HR: 1.15 for DBP of 65-60 mmHg; *p* < 0.005). The authors also found that in elderly patients with coexisting CAD, uncontrolled lowering of the DBP was associated with a higher risk of CV events [HR: 1.17 for DBP of 65-60 mmHg (nadir: 70 mmHg); *p* < 0.02] [10, 58] (Table 1).

The Hypertension in the Very Elderly Trial (HYVET) [59] was designed to resolve all uncertainty about the relative benefits and risks of antihypertensive treatment in elderly populations. In the HYVET trial, 3,845 individuals aged 80 years and older (mean age 83.6 years) with a sustained SBP of 160 mmHg or higher were randomized to indapamide at a dose of 1.5 mg or matching placebo. Perindopril 2 mg or 4 mg, or matching placebo, was added if needed to achieve the target BP of 150/80 mmHg [2, 59]. The study was terminated early after a median follow-up of 1.8 years. The authors found that antihypertensive drug therapy significantly decreased the incidence of the primary endpoint (fatal or nonfatal stroke) by 30 % (*p* = 0.06), as well as other endpoints – fatal stroke by 39 % (*p* = 0.05), all-cause mortality by 21 % (*p* = 0.02), death from CV causes by 23 % (*p* = 0.06) and HF by 64 % (*p* < 0.001) [59]. The achieved BP in the active treatment group was 143.5/77.9 mmHg compared with 158.5/83.2 mmHg in the placebo group. The authors did not observe a J-shaped relationship for either SBP or DBP. However, one should note the important limitations of the HYVET that reduce its generalizability to the entire population of very elderly hypertensives, including (1) the inclusion of very healthy elderly persons (only 12 % had a history of CVD), who would likely be insensitive to a J-curve effect of BP reduction, and (2) the target SBP <150 mmHg, which failed to ascertain whether further reduction would have been beneficial for this particular age group [2, 13, 17, 59].

The Japanese Trial to Assess Optimal Systolic Blood Pressure in Elderly Hypertensive Patients (JATOS) [60] compared the effect of moderate intensity SBP reduction (<140 mmHg) with less intense SBP reduction (140–160 mmHg) in 4,418 elderly (age 65–85 years) hypertensive patients. The SBP achieved in these groups differed by about 10 mmHg (135.9/74.8 vs. 145.6/78.1 mmHg, respectively) after 2 years of follow-up. The authors did not observe a significant difference in the primary endpoint (a composite of CVD, cardiac and vascular disease and renal failure) or secondary endpoints (total deaths and safety issues), or a J-shaped relationship between achieved SBP and outcomes, or any added benefits from more aggressive BP reduction [60]. Similar results were obtained in the Valsartan in Elderly Isolated Systolic Hypertension (VALISH) study published in August 2010 [61]. The authors aimed to establish whether strict BP control (<140 mmHg) was superior to moderate control (≥140 mmHg to <150 mmHg) in reducing CV mortality and morbidity in 3,260 elderly patients with isolated systolic hypertension [61]. At 3 years, BP reached 136.6/74.8 mmHg and 142.0/76.5 mmHg, respectively (*p* < 0.001 for both). The overall rate of the primary composite endpoint was 10.6 per 1,000 patient-years in the strict control group and 12.0 per 1,000 patient-years in the moderate one (HR 0.89; *p* = 0.38). Unfortunately, the study was underpowered to definitively answer whether strict control was superior to less stringent BP targets and did not allow an evaluation of the benefits and risks involved with excess BP reduction (i.e., existence of a J-curve) [10, 61].

A J-curve relationship in this group of patients was observed in a retrospective study of veterans aged over 80 years in which the authors found that patients with DBP >89 mmHg and SBP >139 mmHg had lower mortality compared to those with lower pressures. This relationship was observed even after adjustment for demographics, body mass index, antihypertensive medication use and co-morbidities [62]. Very similar results were observed in a subanalysis of the above-mentioned INVEST trial where 2,180 elderly (>80 years) participants were included [63•]. The authors found age-dependent J-shaped relationships between on-treatment SBP and DBP and the primary outcome (all-cause mortality, nonfatal MI or nonfatal stroke). The SBP nadir increased with increasing age from 110 mmHg in the patients below 60 to 135 mmHg in the 60 to <70 years group to 140 mmHg in patients over 70. The DBP nadir was 75 mmHg for those <60 to <80 years and 70 mmHg for the very old [63•] (Table 1).

The most current studies have also confirmed a need for caution when lowering BP in elderly patients. Ogihara et al. investigated whether the J-curve relationship was observed in a large cohort (*n* = 1,500) of elderly Japanese patients (over 60 years) [64]. At 3 years, the authors showed that the relationship between BP and the incidence of CV events revealed that patients with SBP of less than 120 mmHg had a higher incidence of CV events compared with those with an SBP of 120-139 mmHg [64] (Table 1).

## Blood Pressure Targets in LVH Patients

In the subanalysis of the Losartan Intervention For Endpoint reduction in hypertension study (LIFE) [65], the authors evaluated the risk of stroke, MI, CV death, the composite endpoint of these events and all-cause mortality in relation to in-treatment SBP just prior to an event in 9,193 hypertensive patients with LVH randomly assigned to losartan- or atenolol-based treatment. Patients with in-treatment SBP ≤130 mmHg and SBP between 131 and 141 mmHg were compared with the group with in-treatment SBP ≥142 mmHg [65]. The authors found that SBP ≤130 mmHg was not associated with lower CV risk (in comparison to the group with SBP of 131 to 141 mmHg) and was associated with a significantly increased (29 %) risk of death (HR 1.29, 95 % CI 1.06-1.58) and a trend towards increased CV mortality (HR 1.28, 95 % CI 0.97-1.69) [65]. These outcomes were independent of achieved DBP and treatment modality [65]. Similar to findings in diabetic hypertensive patients, these data do not support treating patients with ECG-LVH to lower SBP goals in order to prevent CVD outcomes and death [10, 13, 17] (Table 1).

Similar results were obtained in our trials [66•, 67]. In the analysis of the Digitalis Investigation Group (DIG) trial [66•], we studied the impact of baseline SBP on outcomes in patients with mild to moderate chronic systolic and diastolic HF. During 5 years of follow-up, all-cause mortality occurred in 35 % and 32 % of matched patients with SBPs ≤120 and >120 mmHg, respectively. HRs for CV and HF mortalities associated with SBP ≤ 120 mmHg were 1.15 (*p* = 0.031) and 1.30 (*p* = 0.006). CV hospitalization occurred in 53 % and 49 % of matched patients with SBPs ≤120 and >120 mmHg, respectively (HR 1.13, *p* = 0.008). HRs for all-cause and HF hospitalizations associated with SBP ≤120 mmHg were 1.10 (*p* = 0.017) and 1.21 (*p* = 0.002) [66•]. Similar results were observed in the *post-hoc* analysis of the Beta-Blocker Evaluation of Survival Trial (BEST) [67] in patients with chronic HF. At 4-year follow-up, HF hospitalization occurred in 38 % and 32 % of patients with SBP ≤120 and >120 mmHg, respectively (HR 1.33; *p* = 0.023) and all-cause mortality in 28 % and 30 %, respectively (HR 1.13; *p* = 0.369). On the basis of the results of these two studies [66•, 67], we concluded that we should be careful when lowering the SBP below 120 mmHg in patients with chronic HF, as it might be connected with adverse CV events (Table 1).

## Conclusions

Randomized controlled trials of antihypertensive treatment provide strong evidence for J-shaped relationships between both DBP and SBP and main outcomes (all-cause mortality, CV mortality, nonfatal and fatal MI, HF, stroke) in the general population of hypertensive patients, as well as in high-risk populations, including patients with CAD, DM and LVH, as well as elderly subjects. Data are also available on a possible J-curve phenomenon in patients with chronic kidney disease (CKD) [47, 48, 68–71] (Table 1) and after stroke/TIA (as a result of secondary prevention therapy) [72, 73, 74•].

Despite many studies in which a J-shaped relationship was observed, the discussion on the BP J-curve phenomenon continues. Some authors still dispute the existence of this phenomenon, claiming among other things that there is nothing indicating a J-curve mechanism in the ACCORD BP study [44••], and they suggest that the findings in the INVEST [36, 43••], VALUE [30], ONTARGET [32, 49] and other trials might be artifacts caused by the observational nature of these analyses [10]. It is also emphasized that the BBB [26], HOT [27], ACCORD BP [44••] and TNT [37] studies were not originally powered to detect harmful effects or non-linear relationships between BP and CV events [10, 75, 76]. Finally, most of the current evidence supporting the J-curve concept comes from *post hoc* analyses that are subject to confounding and have obvious limitations: (1) randomization is lost, (2) the numbers of patients and events in the lowest SBP/DBP group are very small, and (3) patients in the lowest BP groups markedly differ from those with higher BP and often are at increased baseline CV risk [10, 75]. Although these baseline disparities are usually adjusted by sophisticated statistics, it is important to remember that statistics cannot entirely correct large between-group differences [75]. Finally, the nadir should be similar in different studies, at least for patients at a comparable level of CV risk, and a recent review of available data has found widely different nadirs, ranging from 110 to 169 mmHg for SBP and from 55 to 94 mmHg for DBP [10, 13, 17, 75], although most analyses point toward a nadir of 130–140/70–85 mmHg.

Therefore, further studies are still necessary and might contribute to the clarification of this issue, especially with exploration of the J-curve as their primary outcome, with clear and sufficiently large differences in BP levels; these will be able to answer many questions, including the effects of individual antihypertensive agents on the J-curve, the presence or absence of possible confounding factors, or the potential reversibility of the J-curve [10, 13]. Perhaps only the Systolic Blood Pressure Intervention Trial (SPRINT) [77], the results of which are expected to be available in 2018, and Optimal Blood Pressure and Cholesterol Targets for Preventing Recurrent Stroke in Hypertensives (ESH-CHL-SHOT) [74•], which starts recruiting patients in autumn of this year, will be able to finally answer all these questions.

According to the current state of of knowledge, it seems reasonable to suggest lowering of BP to values within the range 130–139/80–85 mmHg, and possibly close to the lower values in this range, in all hypertensive patients (according to current ESH 2009 guidelines) and to be very careful with (and possibly to avoid) further BP reduction, especially in high-risk hypertensive patients [6, 10, 13, 75–81].

## References

[1] Lloyd-Jones D, Adams R, Carnethon M (2009). Heart disease and stroke statistics - 2009 update: a report from the American Heart Association Statistics Committee and Stroke Statistics Subcommittee. Circulation.

[2] Banach M, Aronow WS. Hypertension therapy in the older adults-do we know the answers to all the questions? The status after publication of the ACCF/AHA 2011 expert consensus document on hypertension in the elderly. J Hum Hypertens 2012; DOI: 10.1038/jhh.2012.3. 10.1038/jhh.2012.3 22513754

[3] Chockalingam A, Campbell NR, Fodor JG (2006). Worldwide epidemic of hypertension. Can J Cardiol.

[4] Bielecka-Dabrowa A, Aronow WS, Rysz J, Banach M (2011). The Rise and Fall of Hypertension: Lessons Learned from Eastern Europe. Curr Cardiovasc Risk Rep.

[5] Barylski M, Małyszko J, Rysz J, Myśliwiec M, Banach M (2011). Lipids, blood pressure, kidney—what was new in 2011?. Arch Med Sci.

[6] Mancia G, Laurent S, Agabiti-Rosei E, Ambrosioni E, Burnier M, Caulfield MJ, Cifkova R, Clément D, Coca A, Dominiczak A, Erdine S, Fagard R, Farsang C, Grassi G, Haller H, Heagerty A, Kjeldsen SE, Kiowski W, Mallion JM, Manolis A, Narkiewicz K, Nilsson P, Olsen MH, Rahn KH, Redon J, Rodicio J, Ruilope L, Schmieder RE, Struijker-Boudier HA, Van Zwieten PA, Viigimaa M, Zanchetti A (2009). Reappraisal of European guidelines on hypertension management: a European Society of Hypertension Task Force document. J Hypertens.

[7] Banach M, Kjeldsen SE, Narkiewicz K (2010). Editorial. Controversies in hypertension treatment. Curr Vasc Pharmacol.

[8] Messerli FH, Mancia G, Conti CR, Hewkin AC, Kupfer S, Champion A, Kolloch R, Benetos A, Pepine CJ (2006). Dogma disputed: can aggressively lowering blood pressure in hypertensive patients with coronary artery disease be dangerous?. Ann Intern Med.

[9] Reboldi G, Gentile G, Manfreda VM, Angeli F, Verdecchia P (2012). Tight blood pressure control in diabetes: evidence-based review of treatment targets in patients with diabetes. Curr Cardiol Rep.

[10] Banach M, Michalska M, Kjeldsen SE, Małyszko J, Mikhailidis DP, Rysz J (2011). What should be the optimal levels of blood pressure: Does the J-curve phenomenon really exist?. Expert Opin Pharmacother.

[11] Gluba A, Bielecka A, Mikhailidis DP, Wong ND, Franklin SS, Rysz J, Banach M (2012). An update on biomarkers of heart failure in hypertensive patients. J Hypertens.

[12] Kaplan NM (2011). The diastolic J curve: alive and threatening. Hypertension.

[13] Panjrath GS, Chaudhari S, Messerli FH (2012). The J-point phenomenon in aggressive therapy of hypertension: new insights. Curr Atheroscler Rep.

[14] Stewart IM (1979). Relation of reduction in pressure to first myocardial infarction in patients receiving treatment for severe hypertension. Lancet.

[15] Cruickshank JM, Thorp JM, Zacharias FJ (1987). Benefits and potential harm of lowering high blood pressure. Lancet.

[16] D'Agostino RB, Belanger AJ, Kannel WB, Cruickshank JM (1991). Relation of low diastolic blood pressure to coronary heart disease death in presence of myocardial infarction: the Framingham Study. BMJ.

[17] Dudenbostel T, Oparil S (2012). J curve in hypertension. Curr Cardiovasc Risk Rep.

[18] Polese A, De Cesare N, Montorsi P, Fabbiocchi F, Guazzi M, Loaldi A, Guazzi MD (1991). Upward shift of the lower range of coronary flow autoregulation in hypertensive patients with hypertrophy of the left ventricle. Circulation.

[19] Okoński P, Banach M, Rysz J, Barylski M, Irzmański R, Piechota M, Zasłonka J (2006). L-arginine improves hemodynamic function and coronary flow in an experimental model of ischemia-reperfusion injury. Ann Transplant.

[20] Ma J, Qian J, Ge J, Zeng X, Sun A, Chang S, Chen Z, Zou Y (2012). Changes in left ventricular ejection fraction and coronary flow reserve after coronary microembolization. Arch Med Sci.

[21] Williams B, Lindholm LH, Sever P (2008). Systolic pressure is all that matters. Lancet.

[22] Zanchetti A, Grassi G, Mancia G (2009). When should antihypertensive drug treatment be initiated and to what levels should systolic blood pressure be lowered? A critical appraisal. J Hypertens.

[23] Marschner IC, Simes RJ, Keech A (2007). Biases in the identification of risk factor thresholds and J-curves. Am J Epidemiol.

[24] Kalantar-Zadeh K, Block G, Horwich T, Fonarow G (2004). Reverse epidemiology of conventional cardiovascular risk factors in patients with chronic heart failure. J Am Coll Cardiol.

[25] Sarwar N, Gao P, Seshasai SR, Gobin R, Kaptoge S, DiAngelantonio E (2010). Emerging Risk Factors Collaboration. Diabetes mellitus, fasting blood glucose concentration, and risk of vascular disease: a collaborative meta-analysis of 102 prospective studies. Lancet.

[26] Hansson L for the BBB Study Group (1994). The BBB Study: The effect of intensified antihypertensive treatment on the level of blood pressure, side effects, morbidity and mortality in “well-treated” hypertensive patients. Blood Press.

[27] Hansson L, Zanchetti A, Carruthers SG, Dahlöf B, Elmfeldt D, Julius S, Ménard J, Rahn KH, Wedel H, Westerling S (1998). Effects of intensive blood-pressure lowering and low-dose aspirin in patients with hypertension: principal results of the Hypertension Optimal Treatment (HOT) randomised trial. HOT Study Group. Lancet.

[28] Cruickshank JM (1998). Hypertension Optimal Treatment (HOT) trial. Lancet.

[29] Hedner T, Oparil S, Narkiewicz K, Kjeldsen SE (2009). The J-curve phenomenon revisited. Blood Press.

[30] Julius S, Kjeldsen SE, Weber M, Brunner HR, Ekman S, Hansson L, Hua T, Laragh J, McInnes GT, Mitchell L, Plat F, Schork A, Smith B, Zanchetti A (2004). VALUE trial group. Outcomes in hypertensive patients at high cardiovascular risk treated with regimens based on valsartan or amlodipine: the VALUE randomised trial. Lancet.

[31] Messerli FH, Mancia G, Weber MA, Kjeldsen SE, Holzhauer B, Hua TA (2009). Low blood pressure is associated with increased cardiovascular morbidity (J-shaped curve) in treated hypertensive patients with increased cardiovascular risk: The VALUE Randomized Trial. J Am Coll Cardiol.

[32] Sleight P, Redon J, Verdecchia P, Mancia G, Gao P, Fagard R, Schumacher H, Weber M, Böhm M, Williams B, Pogue J, Koon T, Yusuf S (2009). ONTARGET investigators. Prognostic value of blood pressure in patients with high vascular risk in the Ongoing Telmisartan Alone and in combination with Ramipril Global Endpoint Trial study. J Hypertens.

[33] Mancia G, Schumacher H, Redon J, Verdecchia P, Schmieder R, Jennings G (2011). Blood pressure targets recommended by guidelines and incidence of cardiovascular and renal events in ONTARGET. Circulation.

[34] Banach M, Rysz J (2010). Current problems in hypertension and nephrology. Expert Opin Pharmacother.

[35] Verdecchia P, Staessen JA, Angeli F, de Simone G, Achilli A, Ganau A, Mureddu G, Pede S, Maggioni AP, Lucci D, Reboldi G (2009). Cardio-Sis investigators. Usual versus tight control of systolic blood pressure in non-diabetic patients with hypertension (Cardio-Sis): an open-label randomised trial. Lancet.

[36] Pepine CJ, Handberg EM, Cooper-DeHoff RM, Marks RG, Kowey P, Messerli FH, Mancia G, Cangiano JL, Garcia-Barreto D, Keltai M, Erdine S, Bristol HA, Kolb HR, Bakris GL, Cohen JD, Parmley WW (2003). INVEST Investigators. A calcium antagonist vs a non-calcium antagonist hypertension treatment strategy for patients with coronary artery disease. The International Verapamil-Trandolapril Study (INVEST): a randomized controlled trial. JAMA.

[37] Bangalore S, Messerli FH, Wun C, Zuckerman AL, DeMicco D, Kostis JB, LaRosa JC (2009). J-curve revisited: an analysis of the Treating to New Targets (TNT) Trial. J Am Coll Cardiol.

[38] Bangalore S, Messerli FH, Wun CC, Zuckerman AL, DeMicco D, Kostis JB, LaRosa JC (2010). Treating to New Targets Steering Committee and Investigators. J-curve revisited: An analysis of blood pressure and cardiovascular events in the Treating to New Targets (TNT) Trial. Eur Heart J.

[39] Bangalore S, Qin J, Sloan S, Murphy SA, Cannon CP (2010). PROVE IT-TIMI 22 Trial Investigators. What is the optimal blood pressure in patients after acute coronary syndromes?: Relationship of blood pressure and cardiovascular events in the PRavastatin OR atorVastatin Evaluation and Infection Therapy-Thrombolysis In Myocardial Infarction (PROVE IT-TIMI) 22 trial. Circulation.

[40] Dorresteijn JA, van der Graaf Y, Spiering W, Grobbee DE, Bots ML, Visseren FL (2012). Secondary Manifestations of Arterial Disease Study Group. Relation between blood pressure and vascular events and mortality in patients with manifest vascular disease: J-curve revisited. Hypertension.

[41] Bangalore S, Kumar S, Volodarskiy A, Messerli FH. Blood pressure targets in patients with coronary artery disease: observations from traditional and Bayesian random effects meta-analysis of randomised trials. Heart 2012; (in press). 10.1136/heartjnl-2012-301968 22914531

[42] Tryniszewski W, Kuśmierczyk J, Maziarz Z, Goś R, Mikhailidis DP, Banach M, Rysz J, Pesudovs K (2011). Correlation of the severity of diabetic retinopathy and the heart muscle perfusion in patients with type 2 diabetes. J Diabetes Complicat.

[43] Cooper-DeHoff RM, Gong Y, Handberg EM, Bavry AA, Denardo SJ, Bakris GL, Pepine CJ (2010). Tight blood pressure control and cardiovascular outcomes among hypertensive patients with diabetes and coronary artery disease. JAMA.

[44] The ACCORD Study Group (2010). Effects of Intensive Blood-Pressure Control in Type 2 Diabetes Mellitus. N Engl J Med.

[45] Nilsson PM (2010). ACCORD and risk-factor control in type 2 diabetes. N Engl J Med.

[46] Cooper-DeHoff RM, Egelund EF, Pepine CJ (2011). Blood pressure lowering in patients with diabetes—one level might not fit all. Nat Rev Cardiol.

[47] Berl T, Hunsicker LG, Lewis JB, Pfeffer MA, Porush JG, Rouleau JL, Drury PL, Esmatjes E, Hricik D, Pohl M, Raz I, Vanhille P, Wiegmann TB, Wolfe BM, Locatelli F, Goldhaber SZ, Lewis EJ, Collaborative Study Group (2005). Impact of achieved blood pressure on cardiovascular outcomes in the Irbesartan Diabetic Nephropathy Trial. J Am Soc Nephrol.

[48] Schrier RW, Estacio RO, Esler A, Mehler P (2002). Effects of aggressive blood pressure control in normotensive type 2 diabetic patients on albuminuria, retinopathy and strokes. Kidney Int.

[49] Redon J, Mancia G, Sleight P, Schumacher H, Gao P, Pogue J, Fagard R, Verdecchia P, Weber M, Böhm M, Williams B, Yusoff K, Teo K, Yusuf S (2012). ONTARGET Investigators. Safety and efficacy of low blood pressures among patients with diabetes: subgroup analyses from the ONTARGET (ONgoing Telmisartan Alone and in combination with Ramipril Global Endpoint Trial). J Am Coll Cardiol.

[50] Vamos EP, Harris M, Millett C, Pape UJ, Khunti K, Curcin V, Molokhia M, Majeed A (2012). Association of systolic and diastolic blood pressure and all cause mortality in people with newly diagnosed type 2 diabetes: retrospective cohort study. BMJ.

[51] Banach M, Aronow WS (2011). Should we have any doubts about hypertension therapy in elderly patients?: ACCF/AHA 2011 expert consensus document on hypertension in the elderly. Pol Arch Med Wewn.

[52] Global Health Risks. Mortality and burden of disease attributable to selected major risks. World Health Organization 2009:

[53] Athyros VG, Giouleme O, Ganotakis ES, Elisaf M, Tziomalos K, Vassiliadis T, Liberopoulos EN, Theocharidou E, Karagiannis A, Mikhailidis DP (2011). Safety and impact on cardiovascular events of long-term multifactorial treatment in patients with metabolic syndrome and abnormal liver function tests: a post hoc analysis of the randomised ATTEMPT study. Arch Med Sci.

[54] Aronow WS, Banach M (2012). Ten most important things to learn from the ACCF/AHA 2011 expert consensus document on hypertension in the elderly. Blood Press.

[55] Vokó Z, Bots ML, Hofman A, Koudstaal PJ, Witteman JC, Breteler MM (1999). J-shaped relation between blood pressure and stroke in treated hypertensives. Hypertension.

[56] SHEP Cooperative Research Group (1991). Prevention of stroke by antihypertensive drug treatment in older persons with isolated systolic hypertension: final results of the Systolic Hypertension in The Elderly Program. JAMA.

[57] Pastor-Barriuso R, Banegas JR, Damian J, Appel LJ, Guallar E (2003). Systolic blood pressure, diastolic blood pressure, and pulse pressure: an evaluation of their joint effect on mortality. Ann Intern Med.

[58] Staessen JA, Thijisq L, Fagard R, Celis H, Birkenhäger WH, Bulpitt CJ, de Leeuw PW, Fletcher AE, Forette F, Leonetti G, McCormack P, Nachev C, O'Brien E, Rodicio JL, Rosenfeld J, Sarti C, Tuomilehto J, Webster J, Yodfat Y, Zanchetti A (2004). Systolic Hypertension in Europe (Syst-Eur) Trial Investigators. Effects of immediate versus delayed antihypertensive therapy on outcome in the Systolic Hypertension in Europe Trial. J Hypertens.

[59] Beckett NS, Peters R, Fletcher AE (2008). Treatment of hypertension in patients 80years of age or older. N Engl J Med.

[60] JATOS Study Group (2008). Principal results of the Japanese trial to assess optimal systolic blood pressure in elderly hypertensive patients (JATOS). Hypertens Res.

[61] Ogihara T, Saruta T, Rakugi H, Matsuoka H, Shimamoto K, Shimada K, Imai Y, Kikuchi K, Ito S, Eto T, Kimura G, Imaizumi T, Takishita S, Ueshima H (2010). Valsartan in Elderly Isolated Systolic Hypertension Study Group. Target blood pressure for treatment of isolated systolic hypertension in the elderly: Valsartan in Elderly Isolated Systolic Hypertension Study. Hypertension.

[62] Oates DJ, Berlowitz DR, Glickman ME, Silliman RA, Borzecki AM (2007). Blood pressure and survival in the oldest old. J Am Geriatr Soc.

[63] Denardo SJ, Gong Y, Nichols WW (2010). Blood pressure and outcomes in very old hypertensive coronary artery disease patients: an INVEST Substudy. Am J Med.

[64] Ogihara T, Matsuoka H, Rakugi H (2011). Practitioner's trial on the efficacy of antihypertensive treatment in elderly patients with hypertension II (PATE-hypertension II study) in Japan. Geriatr Gerontol Int.

[65] Okin PM, Hille DA, Kjeldsen SE, Dahlöf B, Devereux RB (2012). Impact of lower achieved blood pressure on outcomes in hypertensive patients. J Hypertens.

[66] Banach M, Bhatia V, Feller MA, Mujib M, Desai RV, Ahmed MI, Guichard JL, Aban I, Love TE, Aronow WS, White M, Deedwania P, Fonarow G, Ahmed A (2011). Relation of baseline systolic blood pressure and long-term outcomes in ambulatory patients with chronic mild to moderate heart failure. Am J Cardiol.

[67] Desai RV, Banach M, Ahmed MI, Mujib M, Aban I, Love TE, White M, Fonarow G, Deedwania P, Aronow WS, Ahmed A (2010). Impact of baseline systolic blood pressure on long-term outcomes in patients with advanced chronic systolic heart failure (insights from the BEST trial). Am J Cardiol.

[68] Norris K, Bourgoigne J, Gassman J, Hebert L (2006). Cardiovascular outcomes in the African American Study of Kidney Disease and Hypertension AASK (Trial). Am J Kidney Dis.

[69] Lewis EJ, Hunsicker LG, Clarke WR (2001). Renoprotective effect of the angiotensin-receptor antagonist irbesartan in patients with nephropathy due to type 2 diabetes. N Engl J Med.

[70] Aronow WS (2012). What should the optimal blood pressure goal be in patients with diabetes mellitus or chronic kidney disease?. Arch Med Sci.

[71] Agarwal R (2009). Blood pressure components and the risk for end-stage renal disease and death in chronic kidney disease. Clin J Am Soc Nephrol.

[72] Piotrowski G, Banach M, Gerdts E, Mikhailidis DP, Hannam S, Gawor R, Stasiak A, Rysz J, Gawor Z (2011). Left atrial size in hypertension and stroke. J Hypertens.

[73] Law MR, Morris JK, Wald NJ (2009). Use of blood pressure lowering drugs in the prevention of cardiovascular disease: meta-analysis of 147 randomised trials in the context of expectations from prospective epidemiological studies. BMJ.

[74] • Optimal Blood Pressure and Cholesterol Targets for Preventing Recurrent Stroke in Hypertensives (ESH-CHL-SHOT). ClinicalTrials.gov Identifier: NCT01563731. *The first study that aimed to investigate the optimal blood pressure levels in patients after stroke/TIA (as well as the J-shaped relationship in this group of patients)*.

[75] Zanchetti A (2010). Blood pressure targets of antihypertensive treatment: up and down the J-shaped curve. Eur Heart J.

[76] Athyros VG, Hatzitolios AI, Karagiannis A, Savopoulos C, Katsiki N, Tziomalos K, Papagianni A, Kakafika A, Gossios TD, Mikhailidis DP, IMPERATIVE Collaborative Group (2011). IMproving the imPlemEntation of cuRrent guidelines for the mAnagement of major coronary hearT disease rIsk factors by multifactorial interVEntion. The IMPERATIVE renal analysis. Arch Med Sci.

[77] Systolic Blood Pressure Intervention Trial (SPRINT). ClinicalTrials.gov Identifier: NCT01206062.

[78] Sethi A, Arora RR (2009). Ambulatory blood pressure as a predictor of cardiovascular risk. Arch Med Sci.

[79] Stepien M, Banach M, Jankowski P, Rysz J (2010). Clinical implications of non-invasive measurement of central aortic blood pressure. Curr Vasc Pharmacol.

[80] Lai HM, Aronow WS, Mercando AD, Kalen P, Desai HV, Gandhi K, Sharma M, Amin H, Lai TM (2012). Risk factor reduction in progression of angiographic coronary artery disease. Arch Med Sci.

[81] Zanchetti A, Mancia G, Black HR, Oparil S, Waeber B, Schmieder RE, Bakris GL, Messerli FH, Kjeldsen SE, Ruilope LM (2009). Facts and fallacies of blood pressure control in recent trials: implications in the management of patients with hypertension. J Hypertens.

